# Administration of Intravenous Furosemide in Patients with Acute Infection: Patient Characteristics and Impact on In-Hospital Outcome

**DOI:** 10.3390/jcm12103496

**Published:** 2023-05-16

**Authors:** Nir Levi, Alon Bnaya, Arik Wolak, Linda Shavit, Sabre Jaffal, Itshak Amsalem, Rafael Hitter, Talya Wolak

**Affiliations:** 1 Internal Medicine Department D, Shaare Zedek Medical Center, Faculty of Medicine, The Hebrew University of Jerusalem, Jerusalem 9112102, Israel; 2 Jesselson Integrated Heart Center, Shaare Zedek Medical Center, Faculty of Medicine, The Hebrew University of Jerusalem, Jerusalem 9112102, Israel; 3 Nephrology Unit, Shaare Zedek Medical Center, Faculty of Medicine, The Hebrew University of Jerusalem, Jerusalem 9112102, Israel

**Keywords:** diuretics, furosemide, mortality, infection

## Abstract

Intravenous (IV) fluid is frequently used to treat patients who have been admitted with an acute infection; among these patients, some will experience pulmonary congestion and will need diuretic treatment. Consecutive admissions to the Internal Medicine Department of patients with an acute infection were included. Patients were divided based on IV furosemide treatment within 48 h after admission. A total of 3556 admissions were included: In 1096 (30.8%), furosemide was administered after ≥48 h, and in 2639 (74.2%), IV fluid was administered within <48 h. Mean age was 77.2 ± 15.8 years, and 1802 (50.7%) admissions were females. In a multivariable analysis, older age (OR 1.01 [95% CI, 1.00–1.01]), male gender (OR 0.74 [95% CI, 0.63–0.86]), any cardiovascular disease (OR 1.51 [95% CI, 1.23–1.85]), congestive heart failure (CHF) (OR 2.81 [95% CI, 2.33–3.39), hypertension (OR 1.42 [95% CI, 1.22–1.67]), respiratory infection (OR 1.38 [95% CI, 1.17–1.63]), and any IV fluid administration (OR 3.37 [95% CI, 2.80–4.06]) were independently associated with furosemide treatment >48 h after hospital admission. In-hospital mortality was higher in patients with furosemide treatment (15.9% vs. 6.8%, *p* < 0.001). Treatment with furosemide in patients admitted with an infection was found to be associated with prolonged hospital stay and increased in-hospital mortality.

## 1. Introduction

Intravenous (IV) fluid administration is a fundamental part of the management of patients with acute infectious disease. Previously published retrospective studies showed that most patients admitted with sepsis and septic shock received early and aggressive treatment with IV fluids [1,2]. However, multiple studies, including high-quality randomized controlled studies, that examined a protocol-based approach to early and goal-directed IV fluid treatment in patients with sepsis and septic shock have demonstrated mixed results [3,4,5,6]. The 2021 Surviving Sepsis Campaign International Guidelines for the Management of Sepsis and Septic Shock suggest the administration of at least 30 mL/kg of IV crystalloid fluid within the first three hours of treatment (weak recommendation, low-quality evidence) [7].

Fluid overload is a potential and possibly serious complication of treatment with IV fluids in patients with sepsis and septic shock and mandates the clinician to repeatedly assess volume status and development of related complications, especially in older patients and in those with comorbidities. According to previous studies, fluid overload is associated with prolonged hospitalization in the intensive care unit (ICU), extended hospital stay, and higher rates of acute kidney injury and mortality rates [8,9,10,11]. This condition often mandates the initiation of treatments aimed to induce negative fluid balance and treat associated complications (e.g., pulmonary congestion and anasarca). Treatment with loop diuretics is common among patients with sepsis and fluid overload in the ICU, and multiple studies have reported patterns of use and patient outcome [12,13,14]. However, data regarding patterns of use, predictors, and patient outcome in patients admitted to the Internal Medicine Department is lacking.

The aim of this study is to determine the characteristics, predictors, and in-hospital outcomes, including all-cause mortality rates of patients admitted to the Internal Medicine Department with acute infectious disease and treated with IV furosemide > 48 h after admission.

## 2. Materials and Methods

### 2.1. Design and Study Population

This is an observational retrospective study. The study population consisted of all consecutive admissions to the Internal Medicine Department at the Shaare Zedek Medical Center between 1 July and 31 December 2019, of adult patients with a primary diagnosis of any acute infectious disease. Exclusion criteria included age <18 years old, pregnancy, chronic treatment with hemodialysis or peritoneal dialysis, and death during the first 48 h of hospital stay. The diagnosis of acute infectious disease and type of infection was based primarily on the clinical judgment of the admitting physician during admission to the Internal Medicine Department. Admissions were divided into two groups based on the need for IV furosemide treatment >48 h after hospital admission (i.e., “IV furosemide” and “non-IV furosemide” groups). The decision to administer IV furosemide was made by the treating physician during the time of drug administration based on clinical indication.

This study complies with the Declaration of Helsinki and has been approved by the Ethics Committee at the Shaare Zedek Medical Center. Participants’ informed consent was waived due to the retrospective design of the study.

### 2.2. Data Collection

All admissions were analyzed for baseline characteristics, baseline medical treatment, type of infection, vital sign measurements during hospitalization, and type of IV fluid if administered. In addition, the length of hospital stay and rates of all-cause in-hospital mortality were examined. In patients with more than one admission event during the study period, only the first event was included in the in-hospital mortality analysis. All data were retrieved from Shaare Zedek Medical Center medical records.

### 2.3. Statistical Analysis

For all ratio variables, means and standard deviations were calculated, and for all nominal variables, absolute frequencies and percentages were calculated. Chi-square with Yates (continuity correction) for independency was carried out between categorical variables. To test the differences between the IV furosemide and the non-IV furosemide, a *t*-test was carried out. A multivariable logistic regression was applied to identify independent predictors for the administration of IV furosemide during hospitalization and it included the administration of any IV fluids, administration of IV hypotonic solution, admission for respiratory infection, hypertension, congestive heart failure (CHF), cardiovascular disease, first oxygen saturation, male gender, and older age. The criterion for significance was alpha (α) = 0.05. Analyses were carried out using SPSS Statistics for Windows, Version 21.0. (IBM Corp, Armonk, NY, USA).

## 3. Results

### 3.1. Baseline Characteristics

As shown in Table 1, of the 3556 admissions that were included in the final analysis, in 1096 (30.8%), IV furosemide was administered > 48 h after hospital admission. Patients in the IV furosemide group were older and more often female when compared to patients in the non-IV furosemide group. When compared to patients in the non-IV furosemide group, patients in the IV furosemide group were more likely to have CHF, diabetes mellitus (DM), and hypertension. In a total of 2639 (74.2%) admissions, IV fluids were administered within <48 h of admission. Patients in the IV furosemide group were more likely to receive IV fluids when compared to patients in the non-IV furosemide group (77.5% vs. 72.8%, respectively; *p* = 0.004). When examining the type of IV fluids, patients in the IV furosemide group tended to receive more hypotonic solutions than isotonic solutions when compared to patients in the non-IV furosemide group (35.6% vs. 16.5% and 73.8% vs. 70.5%, respectively). In addition, patients in the IV furosemide group were significantly more likely to receive ventilatory support (invasive or non-invasive) during their hospital course.

As shown in Table 2, when examining the type of infection for which the patient was admitted to the Internal Medicine Department, patients in the IV furosemide group were significantly more likely to have respiratory and abdominal infections (38.9% vs. 29.5% and 8.1% vs. 5.8%, respectively), while patients in the non-IV furosemide group were more likely to have urogenital infections (20.2% vs. 25.6%). * From 1 January 2016 to 31 December 2019.

When compared to patients in the non-IV furosemide group, patients who received IV furosemide had a higher heart rate on the last measurement during hospitalization, lower systolic and diastolic blood pressure on the last measurement, and lower oxygen saturation on the first and last measurements (Table 3). Although statistically significant, these differences were clinically negligible.

### 3.2. Predictors for IV Furosemide Administration

**Figure 1:**
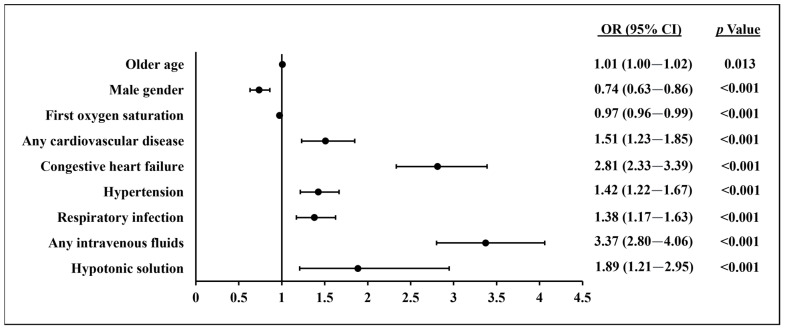
Multivariable analysis for intravenous furosemide administration >48 h after hospital admission. CI, confidence interval; OR, odds ratio.

In a multivariable analysis (Figure 1), independent predictors for the administration of IV furosemide >48 h after hospital admission for an acute infectious disease were found to be older age, low first oxygen saturation, any cardiovascular disease, CHF, hypertension, respiratory infection, any IV fluid administration, and IV hypotonic fluid administration, while males were less likely to receive IV furosemide. All were statistically significant.

### 3.3. Length of Hospitalization and In-Hospital Mortality

**Figure 2:**
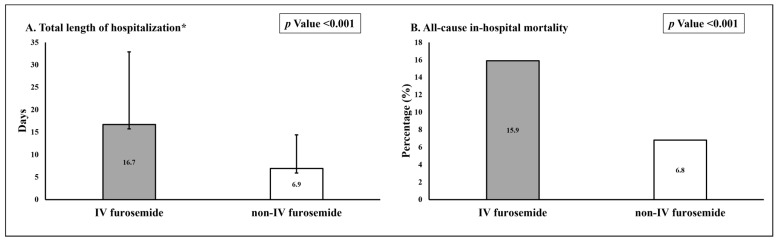
Total length of hospitalization (**A**) and rates of all-cause in-hospital mortality (**B**). * mean ± standard deviation.

The mean length of hospitalization of patients in the IV furosemide group was significantly longer when compared to patients in the non-IV furosemide group (16.7 ± 16.2 vs. 6.9 ± 7.5 days, respectively) (Figure 2A), and rates of all-cause in-hospital mortality were significantly higher in these patients (15.9% vs. 6.8%, respectively) (Figure 2B).

## 4. Discussion

In this study, we aimed to evaluate the prevalence, outcome, and predictors for the treatment with IV furosemide in patients admitted to the Internal Medicine Department with acute infectious disease. We showed that almost one-third of the patients that were admitted with a diagnosis of infectious disease were treated with IV furosemide and that this treatment was associated with significantly prolonged hospital stay and higher rates of all-cause in-hospital mortality. In hospitalized patients, the most common indications for the administration of IV furosemide are fluid overload complications (e.g., anasarca and pulmonary congestion) [15,16]. Hence, we believe that most, if not all, patients treated with IV furosemide in this cohort had signs and symptoms of fluid overload.

Multiple studies have evaluated the effect of IV furosemide in ICU patients [9,17,18,19]. However, to the best of our knowledge, this is the first study to evaluate the effect of IV furosemide in patients admitted to the Internal Medicine Department. Not surprisingly, and in agreement with previous studies, we showed that IV furosemide treatment was more common in patients with a known cardiovascular disease or in those with cardiovascular risk factors (e.g., older age, DM, and hypertension) [18,19]. We also showed that females were significantly more likely to receive IV furosemide during their hospital course. A possible explanation for this finding is that volume status assessment of women admitted with a diagnosis of infectious disease is challenging, which is in part due to differences in physiology and total body water content when compared to males; hence, women might develop fluid overload-related complications. We believe that the physiological differences between the genders should be considered when administering IV fluid treatment in these patients. Further research is needed to clarify these differences and validate practical tools for this aim.

Herein, we showed that patients admitted to the Internal Medicine Department due to respiratory infections were more likely to receive IV furosemide when compared to other infection types, which is possibly due to the similarity between the signs and symptoms of respiratory infection and those of fluid overload complications (e.g., pulmonary congestion). A possible strategy to mitigate these diagnostic and therapeutic challenges may be the use of additional tools (e.g., bed-side echocardiographic measurements and passive leg raise test) for a better assessment of the patient’s hemodynamic and volume status when deciding on treatment with IV furosemide, as previous studies demonstrated the high efficacy of these measures [20,21,22,23].

While in our study, the administration of IV furosemide was associated with a significantly higher in-hospital mortality rate, the association between furosemide administration and short-term mortality in previous studies among ICU patients was inconsistent, but generally, these studies showed that the prognosis of ICU patients treated with IV furosemide was favorable [13,18]. For instance, in their study among patients admitted to the ICU, Zhao et al. showed that treatment with furosemide was associated with lower in-hospital mortality (HR 0.67 [95% CI, 0.60–0.74]) [19]. The discordance between previous studies and our study might be explained, in part, by different patient populations as the above-mentioned studies were conducted on ICU patients with infectious and non-infectious primary diagnoses (e.g., acute respiratory distress syndrome and surgical etiologies). In addition, it is possible that the close monitoring and follow-up available in the ICU allow more accurate management of patient’s volume, while the limited monitoring and follow-up options available in the Internal Medicine Department result in less optimal patient volume management and a poorer outcome.

The above-presented findings should be limited given the observational, retrospective, and single-center design of this study. In addition, data regarding the total dosage and protocol of administration (i.e., bolus versus continuous infusion) of IV furosemide and fluids that were administered during hospital stay were not available, and significant inter-patient variability is likely. In addition, specific indications for furosemide were not defined in the inclusion criteria of this study; hence, it is possible that in a few cases, IV furosemide was administered for indications other than fluid overload complications (e.g., forced diuresis for the treatment of hypercalcemia or rhabdomyolysis). Moreover, it is possible that thresholds for the administration of IV furosemide for the treatment of fluid overload complications were different among different physicians, which might enhance inter-patient variability. In addition, data regarding fluid balance was not available for many patients. Lastly, the population of patients admitted to the Internal Medicine Department in our hospital is highly heterogeneous, especially in terms of indications for admission and disease severity; it is therefore possible that IV furosemide administration is a surrogate marker for disease severity rather than a contributing factor for the adverse outcome shown in this study.

## 5. Conclusions

In conclusion, treatment with IV furosemide in patients admitted to the Internal Medicine Department for acute infectious disease was found to be associated with prolonged hospital stay and increased all-cause in-hospital mortality. Predictors for treatment with IV furosemide during hospitalization for infectious disease were found to be female gender, older age, any cardiovascular disease, CHF, hypertension, respiratory infection, and any IV fluid administration. Further research is needed to determine the effect of furosemide treatment on the clinical outcome of patients with acute infectious disease and volume overload.

## Figures and Tables

**Table 1:** Baseline Patient Characteristics.

Variable	Total N = 3556 (100%)	IV Furosemide N = 1096 (30.8%)	Non-IV Furosemide N = 2460 (69.2%)	*p* Value
Age, mean ± SD (years)	77.2 ± 15.8	80.5 ± 12.7	75.7 ± 16.8	<0.001
Female	1802 (50.7%)	617 (56.3%)	1185 (48.2%)	<0.001
Total no. of hospitalizations * mean ± SD	2.9 ± 2.5	3.2 ± 2.8	2.8 ± 2.4	<0.001
Congestive heart failure	1069 (30.1%)	542 (49.5%)	527 (21.4%)	<0.001
Diabetes mellitus	1388 (39.0%)	457 (41.7%)	931 (37.8%)	0.03
Hypertension	1749 (49.2%)	623 (56.8%)	1126 (45.8%)	<0.001
Malignancy (past/active)	279 (7.8%)	80 (7.3%)	199 (8.1%)	0.458
Intravenous fluids administration	2639 (74.2%)	849 (77.5%)	1790 (72.8%)	0.004
Isotonic solution	2543 (71.5%)	809 (73.8%)	1734 (70.5%)	0.047
Hypotonic solution	796 (22.4%)	390 (35.6%)	406 (16.5%)	<0.001
Ventilatory support	163 (4.6%)	112 (10.2%)	51 (2.1%)	<0.001

**Table 2:** Type of Infection.

Variable	Total N = 3556 (100%)	IV Furosemide N = 1096 (30.8%)	Non-IV Furosemide N = 2460 (69.2%)	*p* Value
Respiratory	1151 (32.4%)	426 (38.9%)	725 (29.5%)	<0.001
Abdominal	231 (6.5%)	89 (8.1%)	142 (5.8%)	0.01
Central nervous system	44 (1.2%)	15 (1.4%)	29 (1.2%)	0.758
Urogenital	850 (23.9%)	221 (20.2%)	629 (25.6%)	<0.001
Skin and soft tissues	1146 (32.2%)	356 (32.5%)	790 (32.1%)	0.859
Endovascular	21 (0.6%)	11 (1.0%)	10 (0.4%)	0.06
Other	11 (0.3%)	4 (0.4%)	7 (0.3%)	-

**Table 3:** Vital Sign Measurements during Hospitalization.

Variable	Total N = 3556 (100%)	IV Furosemide N = 1096 (30.8%)	Non-IV Furosemide N = 2460 (69.2%)	*p* Value
Heart rate—first	87.7 ± 25.0	86.7 ± 25.7	88.1 ± 24.7	149
Heart rate—last	72.6 ± 21.5	74.1 ± 22.2	72.0 ± 21.2	0.08
Systolic blood pressure (mmHg)—first	129.2 ± 29.7	128.4 ± 29.7	129.5 ± 29.7	0.355
Systolic blood pressure (mmHg)—last	125.8 ± 25.1	123.2 ± 25.4	126.9 ± 24.9	<0.001
Diastolic blood pressure (mmHg)—first	72.8 ± 20.4	72.2 ± 29.7	73.1 ± 14.6	0.268
Diastolic blood pressure (mmHg)—last	71.1 ± 12.5	69.4 ± 12.6	71.6 ± 12.4	<0.001
Oxygen saturation (%)—first	92.2 ± 6.0	91.3 ± 7.3	92.7 ± 5.3	<0.001
Oxygen saturation (%)—last	93.0 ± 4.5	92.5 ± 5.2	93.2 ± 4.1	<0.001
Temperature (°C)—first	37.06 ± 0.8	37.0 ± 0.8	37.1 ± 0.8	<0.001
Temperature (°C)—last	36.7 ± 0.4	36.7 ± 0.5	36.7 ± 0.4	0.432

## Data Availability

Data are unavailable due to privacy and ethical restrictions.
